# A man with culture sterile cavitating lung lesions: molecular techniques can provide the key to diagnosis

**DOI:** 10.1099/acmi.0.000510.v3

**Published:** 2023-09-01

**Authors:** Ranvir S. P. Cheema, Prabhjoyt K. Kler, Alpha Madu, Joanna Macve, Andrew MacDuff

**Affiliations:** ^1^​ Department of Critical Care, New Cross Hospital, The Royal Wolverhampton NHS Trust, Wolverhampton Road, WV10 OQP, UK; ^2^​ Department of Microbiology, Black Country Pathology Services, The Royal Wolverhampton NHS Trust, Wolverhampton Road, Wolverhampton, WV10 OQP, UK

**Keywords:** 16S rDNA PCR, atypical pneumonia, case report, community acquired pneumonia, lung biopsy

## Abstract

A 20-year-old male presented to the Emergency Department with pyrexia, dyspnoea, chest pain and haemoptysis. Cavitating lung lesions were noted on chest X-ray and the patient was admitted to the intensive care unit where he was intubated and ventilated. Routine investigations including serial cultures did not provide an aetiological diagnosis. As such, a CT-guided lung biopsy was carried out and 16S rDNA PCR was undertaken on the sample. This identified *Fusobacterium necrophorum* as the causative organism. The patient was treated for Lemierre’s syndrome and successfully discharged from hospital. This case highlights how DNA tissue typing on a lung biopsy sample can be the key to successful diagnosis in an atypical pneumonia and raises the question as to whether this infrequently used approach should be added to forthcoming community acquired pneumonia guidelines.

## Data Summary

No new data, tools, software or code has been generated or is required for our work to be reproduced.

## Dr Ranvir Cheema, ICU Senior House Officer (SHO), CT2 ACCS anaesthetics

A 20-year-old male Kurdish college student with no past medical history presented to the emergency department with a 1 week history of fever, shortness of breath, cough, chest pain, and haemoptysis. He reported that he was convalescing from a SARS-CoV-2 infection, which he had acquired 2 weeks earlier and that his symptoms had been improving until a recent deterioration. He denied any significant weight loss. He was a current smoker, smoking 20 cigarettes a day for the last 7 years. He had relocated to England from Iran a year earlier; he denied any other recent travel history. He kept a pet cockatoo. He denied being sexually active or any recreational drug use.

Physical examination revealed an acutely ill-looking, cachectic, dyspnoeic, and diaphoretic young man. He was noted to be jaundiced. Bronchial breath sounds were heard bilaterally, and his oxygen saturation was 90 % on high flow oxygen delivered through a non-rebreathing mask at 15 Lpm. His pulse rate was 120 beats/minute (sinus rhythm), and his blood pressure was 119/54 mmHg. Heart sounds were normal and capillary refill time was less than 2 s. Swelling of his left index finger and first metacarpophalangeal joint was noted. No neck swelling was noted. Abdominal examination was unremarkable, and no rash or limb swelling was noted. He was not confused.

Arterial blood analysis revealed type one respiratory failure (pH 7.41, PaO_2_ 11, PaCO_2_ 4.8). He was admitted into the intensive care unit (ICU) due to the high oxygen requirements and he was commenced on oxygen supplementation with high flow nasal oxygen, requiring a FiO_2_ of 70 % and flow rate of 50 Lpm in order to achieve adequate arterial oxygen saturation. Empirical antibiotics for presumed lower respiratory tract infection were commenced with a combination of piperacillin-tazobactam and metronidazole for broad spectrum coverage.

## Dr Prabhjoyt Kler, ICU Registrar, ST8 anaesthetics/ Intensive Care Medicine

His initial blood tests showed raised inflammatory markers with a white cell count of 36×10^9^ l^−1^ and C-reactive protein (CRP) of 360 mg l^−1^. He was also thrombocytopenic (platelet count of 82×10^9^ l^−1^). His bilirubin was raised (147 µmol l^−1^), INR was 1.4, ALT was 69 IU l^−1^, and ALP was 108 IU l^−1^. His renal function was normal. He was hyponatraemic (serum sodium level: 121 mmol l^−1^). COVID-19 PCR test was negative.

**Fig. 1.**
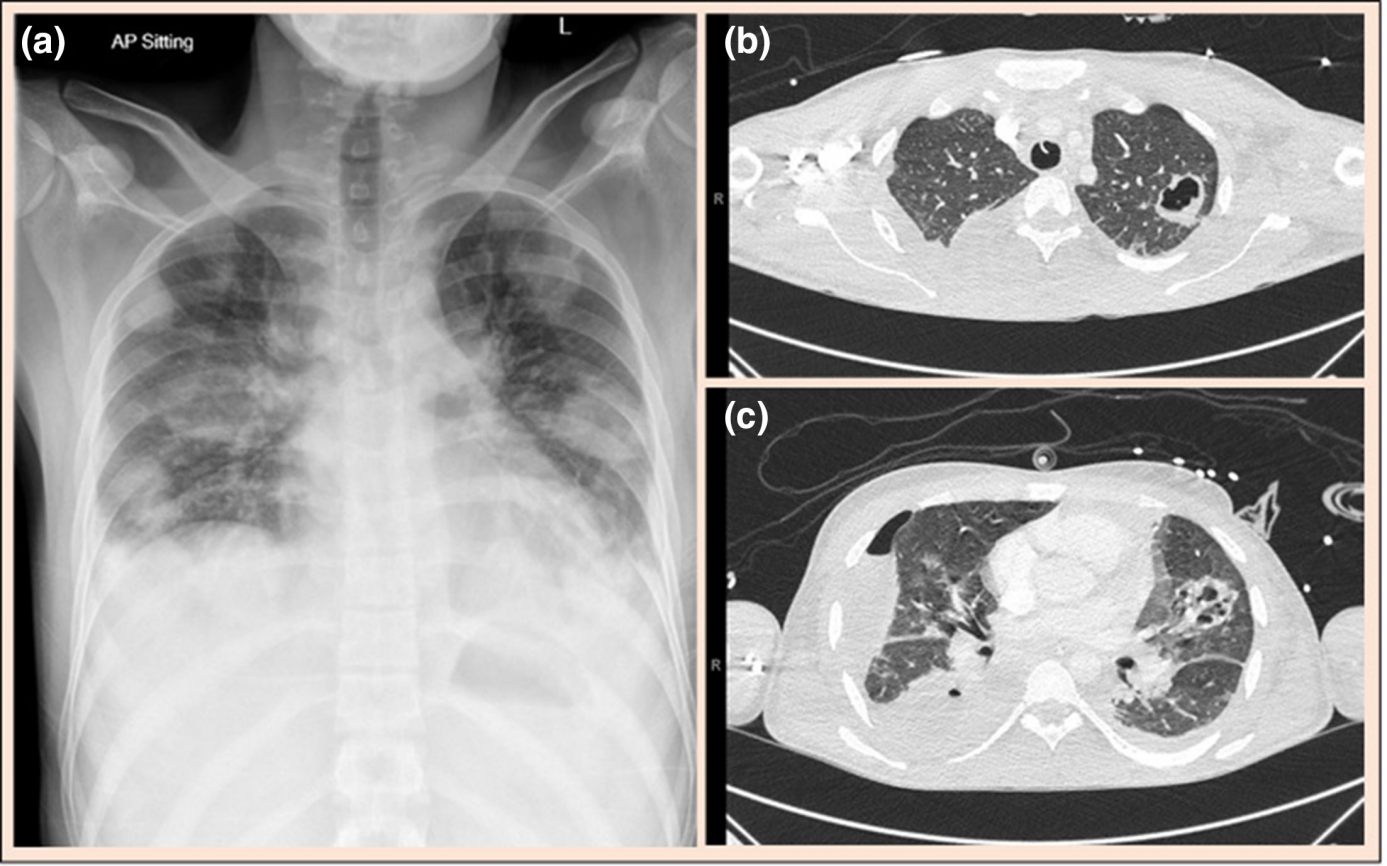
Chest X-ray (**a**) and axial CT slices (**b and c**) demonstrating multiple cavitary lesions present in both lungs.

His chest X-ray showed multiple bilateral cavitary lesions, Fig. 1a. The differential diagnoses at this point were a lower respiratory tract infection possibly caused by *Staphylococcus aureus* or atypical organisms, pulmonary tuberculosis, or a non-infectious process such as metastatic pulmonary lesions, or a vasculitis. Testicular examination, to exclude a primary testicular tumour, was normal. No other features suggestive of a malignancy were noted on history or examination.

A CT scan of his thorax, abdomen and pelvis confirmed the presence of multiple lung lesions with early cavitation, Fig. 1b, c. Bi-basal consolidation and collapse, bilateral pleural effusions, and hepatosplenomegaly were also noted. There was no mediastinal lymphadenopathy. A conclusive radiological diagnosis was not achieved thus a broad range of differentials were proffered; infective pathologies such as multifocal bacterial pneumonia, fungal pneumonia, and atypical tuberculosis, and autoimmune diseases such as autoimmune granulomatosis with polyangiitis.

A vasculitis screen and autoimmune serology were requested and returned as negative. HIV, acid fast bacilli smears, cultures, and viral hepatitis serology were also negative. Pneumococcal and Legionella urine antigens were negative. Serial blood culture samples returned as sterile. We carried out a bronchoalveolar lavage and sent the fluid sample for culture. This also returned sterile. At this point we considered infective endocarditis as one of the possible diagnoses, however no vegetations were noted on transthoracic echocardiogram.

## Dr Ranvir Cheema, ICU SHO

Despite treatment with antibiotics and oxygen supplementation, the patient’s oxygen requirement and work of breathing continued to deteriorate. He was intubated on the second day of admission. His antimicrobial therapy was changed to piperacillin-tazobactam and linezolid, to cover the possibility of Panton-Valentine Leucocidin-producing *Staphylococcus aureus *.

## Dr Prabhjoyt Kler, ICU registrar

Intercostal drains for bilateral pleural effusions were inserted on his third day of admission. One litre of blood-stained fluid was drained from each side and pleural fluid analysis was suggestive of a transudate; cytology was negative for malignant cells and indicative of a parapneumonic effusion.

By the eighth day of admission, we still did not have a definitive diagnosis despite extensive investigations. We repeated a CT scan of the thorax and on the advice of the microbiologists, conducted a CT-guided lung biopsy. The CT scan showed progression of the lung lesions with further cavitations and persistent bilateral pleural effusions. Histology from the biopsy confirmed an acute inflammatory process in keeping with an abscess. The tissue culture also returned sterile.

With no clear diagnosis, a broad range 16S rDNA was requested on the pleural fluid; this was negative.

## Dr Ranvir Cheema, ICU SHO

Following improvements in respiratory parameters, he was successfully extubated on the eleventh day of admission and intercostal drains were removed. Inflammatory markers had not significantly improved (CRP 245 mg l^−1^) and he remained pyrexial, thus antibiotic therapy was escalated to meropenem, doxycycline and metronidazole.

## Dr Prabhjoyt Kler, ICU registrar

By the eighteenth day and under the guidance of the microbiologists, a 16S rDNA PCR was requested on the lung biopsy sample and this identified *Fusobacterium necrophorum* pointing towards a diagnosis of Lemierre’s syndrome (LS). A subsequent bedside ultrasound scan of the neck showed a thrombus in the right internal jugular vein (IJV), consolidating the diagnosis.

## Dr Joanna Macve, microbiology consultant and Dr Ranvir Cheema, ICU SHO

Broad range 16S rDNA sequencing has become more available in the last two decades, with the widespread use of PCR and DNA sequencing. In the UK it is not available in most hospital microbiology laboratories, but samples can be sent to the reference laboratories for testing. It has particularly helped in rapidly and accurately identifying bacteria with unusual phenotypic profiles, that are rare, slow-growing or non-viable, causing culture-negative infections [1]. The 16S ribosomal subunit is present in all bacteria and is encoded for by the 16S rDNA gene. It contains highly conserved regions, separated by variable sequences. Primers targeted at the conserved regions are used to amplify the bacterial genome, which then allows sequencing of the variable regions to be carried out. This method has a significant advantage over routine cultures, in that it can detect non-viable DNA, thus can be effectively used in patients who have commenced a prolonged course of antimicrobial therapy. It is being increasingly used for aetiological diagnosis in culture-negative infective endocarditis; Patel [2] found that this method can prevent delayed diagnosis of LS.

## Dr Alpha Madu, ICU SHO, CT2 ACCS acute medicine

LS is a septic thromboembolic complication of bacterial pharyngotonsillitis that can be fatal if not treated early. It is classically defined as a triad of a deep-seated neck infection and septicaemia, thrombophlebitis of the IJV, and metastatic infections from septic emboli. It usually presents with sore throat and subsequent involvement of the IJV causing thrombophlebitis, culminating in a fulminant multi-systemic organ failure. Multi-organ involvement is usually present by the time of diagnosis [3]. Joint involvement is reported to occur in 16–26 % of cases.

About two-thirds of LS cases are caused by *Fusobacterium necrophorum *, a non-spore forming obligate anaerobic Gram-negative bacilli that forms part of the normal flora of the upper respiratory tract in humans and animals. It invades the connective tissue of the pharynx and causes a localised septic thrombophlebitis in the tonsillar veins and IJV. Septic embolization from the IJV thrombophlebitis is responsible for the systemic manifestations of the disease and explains the joint swelling noted on admission.

Pulmonary involvement is the commonest systemic manifestation of the disease and may present as pleuritic chest pain, cough, dyspnoea, or haemoptysis, all of which were present in our patient. Around 10–15 % of patients develop an empyema [3]. A striking feature of LS is the rapid progression of the lung lesions and pleural effusion despite antibiotic therapy as seen in the index case [3]. Some patients develop acute respiratory distress syndrome (ARDS) with less than 10 % requiring mechanical ventilation [3].

LS is rare (one case per one million people per year) [3] hence its diagnosis is challenging. A positive culture helps confirm the diagnosis. Our case was particularly challenging as serial blood cultures as well as tissue and bronchoalveolar lavage (BAL) cultures were all negative. There have been multiple recent case reports of LS thought to be precipitated by COVID-19 infection [4–6]; it is possible that the COVID-19 infection may have provoked LS in this case.

The treatment of LS is with an extended course of antibiotics (minimum of 4 weeks), usually metronidazole and β-lactam antibiotics [3]. Complications of LS occur as a result of the metastatic septic emboli. Mortality rate is as high as 90 % without antibiotic treatment and less than 20 % with early antibiotic therapy. Anticoagulation has not been demonstrated to reduce mortality or increase vessel recanalization [7], however in this case, due to the presence of the IJV thrombus, we chose to anticoagulate the patient with treatment dose low molecular weight heparin whilst he remained an inpatient and this was then converted to a direct oral anticoagulant on discharge.

## Dr Joanna Macve, consultant in microbiology

This case demonstrates the utility of 16S rDNA PCR of tissue samples in aiding diagnosis of infections caused by bacteria that are fastidious or cannot be cultured, or where prior antibiotic use affects organism recovery. Currently the cost and availability mean that it can only be used selectively, but it is vital to keep it in mind when cultures are negative, but infection is strongly suspected.

## Dr MacDuff, consultant in intensive care and respiratory medicine

This was an interesting case of a young man with a multi-system illness presenting with acute respiratory failure. Initial treatment clearly focused on a possible infectious aetiology with broad spectrum antibiotics in line with national guidelines. National (BTS) [8] and international (ATS) [9] guidelines recommend identifying the causative organism if possible. However, here all standard microbiology investigations were unhelpful. Given the diagnostic uncertainty with the possibility of exotic infectious agents or of non-infectious processes (vasculitides, neoplasia) being missed, we proceeded to undertake a lung biopsy, an uncommon procedure in intensive care.

Histopathology was unhelpful and tissue culture was sterile. A definitive diagnosis was only possible by the astute request of 16S rDNA PCR on the sample by our microbiology colleagues. This shows the importance of a strong working relationship between critical care and our microbiology colleagues. We are fortunate to have managed to continue daily microbiology ward rounds during the COVID-19 pandemic and subsequently.

After discharge from the ICU to a respiratory ward, the patient received intravenous antibiotics for a total of 25 days and was discharged home after a 27 day hospital stay on a 3 month course of oral co-amoxiclav.
